# Enhancing Adherence and Mental Well-Being in Pediatric Growth Hormone Therapy: Feasibility Prospective Observational Study of a Family-Centered Digital Companion

**DOI:** 10.2196/67102

**Published:** 2025-10-27

**Authors:** Antonio de Arriba Muñoz, Amalia M García-Durán, Patricia Sanz-Aznar, Silvia Quer-Palomas, Ioannis Bilionis, Alba Xifra-Porxas, Joia Nuñez, Ricardo C Berrios, Luis Fernández-Luque

**Affiliations:** 1 Endocrinology Pediatric Unit, Hospital Universitario Miguel Servet, Zaragoza, Spain; 2 Instituto de Investigación Sanitaria de Aragón, Zaragoza, Spain; 3 Adhera Health Inc, 101 Cooper St, Santa Cruz, CA, United States, 1 8313455357

**Keywords:** growth hormone, hormone therapy, caregiver fatigue, well-being, pediatric, paediatric, digital health, digital intervention

## Abstract

**Background:**

Managing a child undergoing growth hormone treatment (GHt) can be burdensome for the families, which can lead to psychological problems and poor treatment adherence. The Adhera Caring Digital Program (ACDP) is a mobile-based digital health intervention designed to support the physical and mental well-being of families of individuals with chronic conditions.

**Objective:**

This study aimed to evaluate the clinical feasibility of a digital intervention to support families by focusing on caregivers of children undergoing GHt and its impact on treatment adherence.

**Methods:**

This is a prospective observational study. A total of 51 caregivers of children undergoing GHt with low adherence (below 85%) to treatment were recruited at the Pediatric Endocrinology Unit at the Miguel Servet Children’s University Hospital and enrolled into the ACDP for 3 months.

**Results:**

A total of 51 parents participated in the digital intervention for 3 months. The use of ACDP was associated with a significant increase in adherence rate (*P*<.001). At baseline, all families had suboptimal adherence (below 85%), and after the intervention, 75% (n=38) of the families reached optimal levels of adherence. Also, the perceived pain of injection was reduced, as well as anxiety and stress. Initially, 21.56% (n=11) of caregivers reported depression symptoms, categorized as mild (11.76%, n=6), moderate (7.84%, n=4), and extremely severe (1.96%, n=1), while post intervention, only 1.96% (n=1) of caregivers reported depression as “severe.” Anxiety levels at baseline were reported by a total of 23.53% (n=12) of caregivers (mild: 7.84%, n=4, moderate: 13.73%, n=7, and severe: 1.96%, n=1). After the intervention, only 11.76% (n=6) reported mild (5.88%, n=3) or moderate (5.88%, n=3) anxiety levels. Initially, 23.5% (n=12) of caregivers reported stress as mild (7.84%, n=4), moderate (13.72%, n=7), and severe (1.96%, n=1) stress, and following the intervention, these symptoms reduced to 7.84% (n=4) (mild: 5.88%, n=3, severe: 1.96%, n=1).

**Conclusions:**

The ACDP is a promising tool, and it has been demonstrated to significantly increase the adherence rate, adding value to the patient and caregiver journey, and improving the management of growth hormone deficiency while promoting the overall well-being of family caregivers. Our results show that the digital support provided by the solution significantly increased the quality of life of the caregivers by increasing their psychological, emotional, and social well-being and decreasing their depression, anxiety, and stress symptoms.

## Introduction

### Background

Growth hormone (GH) deficiency (GHD) in the pediatric population causes short stature [1]. Children affected by GHD have also been reported to have worse quality of life, cognitive function, and fatigue than those with normal height [2,3]. Short stature in children is also associated with anxiety, depression, and social withdrawal [4-11]. Importantly, recent findings suggest that poor emotional well-being and health-related quality of life (HrQoL) of caregivers and/or parents are also negatively related to children’s HrQoL, particularly in the context of pediatric health conditions.

These children’s families are responsible for managing the disease. Within the family, family caregivers are the ones managing its treatment (daily injections of recombinant human GH) and the children’s mental health. Caring might be burdensome and challenging [12,13], exposing the family caregiver to the risk of developing psychological problems [14]. Indeed, parental stress has been described as one of the consequences of managing GHD in children, leading to poorer adherence to GH therapy (GHt) and an impact on their environment and health [15,16]. Overall, family caregiver fatigue has been found to significantly impact the well-being of the caregiver and the patient living with a chronic condition [17]. However, the impact of the family caregiver specifically on GHDs remains unclear [18,19].

Digital health (DH) solutions are transforming the health care sector, as they can be a cost-effective option that allows accessible family-centered and personalized interventions. Indeed, mobile interventions have been proven effective in supporting caregivers in the management of chronic conditions [20]. One such innovation is the Easypod system, an electronic, fully automated device designed to streamline the GH treatment process. This system provides reliable real-time injection data, enabling health care providers to monitor patients’ progress and make more informed treatment decisions. By identifying nonadherent patients earlier, the Easypod allows for more targeted support, enhancing both treatment adherence and outcomes [21].

### The Adhera Caring Digital Program

The Adhera Caring Digital Program (ACDP) is a comprehensive, digitally delivered program designed to support the physical and mental well-being of family caregivers of individuals with chronic conditions. It aims to improve self-management and health outcomes for both the patient and their family caregiver [22]. The ACDP includes access to a mobile app for family caregivers, which is integrated into an artificial intelligence–powered platform that includes integrated data from injector devices, as well as tools for supporting clinicians to follow up on the health of the families.

This study aimed to evaluate the clinical feasibility of a digital intervention to support families by focusing on caregivers of children undergoing GHt and its impact on treatment adherence.

### Previous Research

GHt for children is a critical yet challenging process that often demands significant effort from family caregivers. The burden of managing GHt can negatively impact the psychological well-being of caregivers and adherence to the treatment protocol, which, in turn, affects the health outcomes of the children undergoing treatment. This challenge necessitates the development of effective support systems for caregivers to ensure better adherence and overall health outcomes.

Recent advancements in digital health interventions have shown promising results in supporting caregivers of children with chronic conditions, including those undergoing GHt. Studies have demonstrated that digital tools can provide substantial benefits by improving treatment adherence and enhancing the mental health of caregivers. For instance, a recent study by Dimitri et al (2021) [23] found that digital interventions could significantly enhance treatment adherence in children with GHDs by providing real-time support and monitoring capabilities.

In another study, Savage et al [13] highlighted the transformative potential of DH platforms in pediatric endocrinology, emphasizing how patient-generated data and interactive support can lead to more integrated and personalized care models. This approach improves not only adherence but also the overall quality of care provided to patients.

The mental health and well-being of caregivers are crucial factors in ensuring successful GHt management. Zhai et al [24] reported the positive impact of DH interventions on reducing caregiver stress and anxiety, which, in turn, improved their ability to manage the treatment regimen effectively [24]. These findings underscore the importance of addressing the psychological needs of caregivers through innovative digital solutions.

Additionally, Lorca-Cabrera et al [25] explored the role of mobile health applications in supporting self-management and emotional well-being among caregivers. Their research showed that these applications could reduce the psychological burden on caregivers, thereby improving their overall quality of life and enhancing their capacity to support their children’s treatment [25].

The ACDP builds upon these foundations by offering a comprehensive digital health intervention designed to support the physical and mental well-being of caregivers managing children undergoing GHt. This study aims to evaluate the clinical feasibility of ACDP and its impact on treatment adherence, contributing to the growing body of evidence supporting the integration of DH solutions in chronic disease management.

## Methods

### Overview

Family caregivers (ie, parents) of children with suboptimal adherence to GHt were recruited to the study at the Pediatric Endocrinology Unit at the Miguel Servet Children’s University Hospital and were provided with access to the ACDP for 3 months. The protocol was prospectively registered in ClinicalTrials.gov (NCT04812665).

Participants were assessed twice (at recruitment and at 3 months’ follow-up). The data collected were (1) demographic data (only at recruitment); (2) positive mood assessed with the Positive and Negative Affect Schedule (PANAS) [26]; (3) distress assessed using the Depression Anxiety and Stress Scale-21 (DASS-21) [27], general well-being assessed using the Mental Health Continuum Short Form (MHC-SF) [28], and self-efficacy assessed using the Generalized Self-Efficacy Scale (GSES) [29]; (4) HrQoL assessed using the KIDSCREEN-10 [30] and the Quality of Life in Short Stature Youth (QoLISSY) [31]; and (5) GHt adherence (%) by Easypod-Connect.

### Recruitment

The inclusion criteria were as follows:

- Adherence to GHt monitored in the last month prior to enrollment indicates a ratio less than 85%.
- Family caregivers (and legal guardians) of children who receive GHt in accordance with approved indications in Spain.
- Explicit agreement on data sharing regarding adherence to GHt gathered through the Easypod-Connect.
- Participants must be able to interact with mobile phones and be willing to install the mobile-based solution of the study in their smartphone.
- Participants must sign the specific informed consent form for the study.

Only one legal guardian per child can participate in the study.

### Procedure

The ACDP is a noninvasive, digitally delivered intervention designed to support family caregivers of children with chronic conditions. Specifically tailored for the context of GHt, the ACDP offers condition-specific educational content, evidence-based caregiving strategies, and self-management tools to help caregivers monitor progress and remain engaged with their child’s treatment. To promote emotional well-being and adherence, the program delivers personalized motivational messages generated by an artificial intelligence–driven health recommender system. This system tailors its recommendations using both objective and patient-reported data: objective adherence data are collected via Easypod-Connect, an electronic auto-injector device that monitors and transmits GH administration data, while patient-reported outcomes are assessed at baseline and at 3-month follow-up using validated psychometric instruments (see Table 1). The ACDP is part of the Adhera Health Precision Digital Companion Platform [32], which has been developed using the best practices regarding data protection and quality management in accordance with the ISO (International Organization for Standardization) 27001 and ISO 13465 guidelines.

**Table 1.** SPIRIT (Standard Protocol Items: Recommendations for Interventional Trials) table.

Procedures	Visit 1 (baseline)	Visit 2 (3 mo)	Digital health intervention period	Follow-up (post intervention)
Screening for inclusion criteria	✓			
Informed consent	✓			
Baseline HrQoL^a^ questionnaire (QoLISSY)^b^	✓			
Emotional well-being assessment (PANAS^c^)	✓			
Self-efficacy (GSES^d^)	✓			
Adherence monitoring		✓	✓ (throughout intervention)	✓
Digital support program usage			✓	
Child’s growth monitoring	✓		✓	
Caregiver’s QoL^e^ follow-up (QoLISSY)		✓		✓

a HrQoL: health-related quality of life.

b QoLISSY: Quality of Life in Short Stature Youth.

c PANAS: Positive and Negative Affect Schedule.

d GSES: Generalized Self-Efficacy Scale.

e QoL: quality of life.

### Statistical Analysis

Participants’ questionnaire answers were digitally recorded through a Microsoft Forms form and extracted to a Microsoft Excel database. Descriptive analysis (means, SDs, and percentages) was used for demographic data. After checking the normality of each variable, pre-post *P* values of the psychometrics’ parameters were checked using the Student *t* and Wilcoxon tests.

### Ethical Considerations

This study protocol was reviewed and approved by the Spanish Ethics Committee C.P.-C.I. PI20/494. Written informed consent was obtained from participants (or their parent/legal guardian/next of kin) to participate in the study.

The privacy and confidentiality of all research participants were strictly maintained; all data were de-identified before analysis, and no personally identifiable information was collected or reported. Participants were not compensated for their participation in this study.

## Results

### Participants’ Characteristics

A total of 65 parents provided informed consent. Of these, 14 were excluded from the analysis, as they did not access the digital intervention. The final sample’s (n=51) characteristics are described in Table 2. Participants were 42.14 (SD 5.78) years old on average, and the sample mostly comprised women (n=35, 68.6%). The average age of the child taken care of was 7.9 (SD 2.9) years, receiving GHt for 36.51 (SD 28.43) months. Regarding the education level, 15 (29.4%) caregivers hold a university degree, 20 (39.2%) have professional training, 12 (23.5%) finished high school, and 4 (7.8%) finished primary school.

**Table 2.** Descriptive characteristics of the sample (N=51).

Characteristic		Value
Caregiver’s gender, n (%)		
Male		16 (31.4)
Female		35 (68.6)
Caregiver’s age (years), mean (SD)		41.45 (7.59)
Caregiver’s marital status, n (%)		
Single		10 (19.6)
Married		33 (64.7)
Divorced		8 (15.7)
Education, n (%)		
Primary education		4 (7.8)
Secondary education/high school		12 (23.5)
Professional training		20 (39.2)
University degree		15 (29.4)
Child’s gender, n (%)		
Male		25 (49.0)
Female		26 (51.0)
Child’s age (years), mean (SD)		7.98 (2.98)
Time under treatment (months), mean (SD)		36.51 (28.43)

### Statistical Analysis

#### Quality of Life of Children

**Figure 1.**
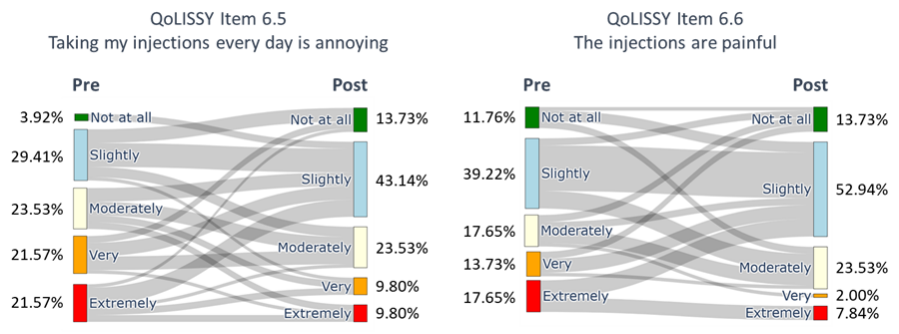
Changes in quality of life in youth with short stature (baseline and 12 wk).

Almost all the psychometric questionnaires’ results showed significant differences (ie, *P*<.05) after using the ACDP for 3 months (Table 3 and Figure 1). General health and quality of life were significantly improved based on KIDSCREEN-10 [30] and QoLISSY [31] psychometrics. General and Social well-being also improved significantly, while emotional well-being has improved as QoLISSY emotional scale scores increased, and the PANAS [26] indicated a statistically significant increase in positive affect (mood) as well as a decrease in negative affect. Social well-being also enhanced significantly according to both MHC-SF [28] and QoLISSY questionnaires. Self-efficacy, assessed using the GSES [29], increased, as did the QoLISSY coping score.

**Table 3.** Comparison of family caregiver characteristics at baseline and 3 months after the intervention

Variable	Baseline	3 months	Statistic	*P* value	Effect size (η^2^)
PANAS^a^					
Positive Affect	30.78±7.27	36.53±8.65	-4.5	<.001	0.115
Negative Affect	18.98±6.02	14.57±4.18	85	<.001	0.154
DASS-21^b^					
Depression Scale	2.98±3.15	1.45±2.10	106	<.001	0.075
Anxiety Scale	2.41±1.95	1.24±1.61	160	<.001	0.098
Stress Scale	5.71±3.15	3.06±3.18	151.5	<.001	0.149
MHC-SF^c^					
General wellbeing	0.62±0.17	0.69±0.21	363	.008	0.037
Emotional wellbeing	0.67±0.20	0.71±0.20	241	.15	0.011
Psychological wellbeing	0.67±0.18	0.72±0.23	454	.17	0.013
Social wellbeing	0.52±0.19	0.65±0.22	-3.7	<.001	0.086
GSES^d^	31.31±5.51	33.43±5.31	336	.04	0.036
KIDSCREEN 10					
HRQoL^e^	49.19±10.82	60.89±16.86	194	<.001	0.146
General Health	3.43±0.81	4.22±0.81	40.5	<.001	0.191
QoLISSY^f^					
HrQoL	74.94±15.93	80.67±14.46	452	.048	0.034
Emotional scale	79.41±15.07	82.35±13.53	442	.28	0.01
Physical scale	75.57±19.51	81.94±18.38	351.5	.04	0.028
Social Scale	69.83±20.35	77.72±18.15	294.5	.02	0.04
Coping scale	40.98±17.25	48.48±21.32	347.5	.01	0.036
Height-related beliefs scale	78.31±25.96	66.30±28.20	208.5	.004	0.047
Treatment scale	53.54±17.77	67.37±21.93	197.5	<.001	0.107
Child’s future scale	73.63±25.57	82.65±17.98	290.5	.04	0.04
Effects of child’s short stature on family scale	68.45±18.94	77.72±18.20	333.5	.009	0.059
Treatment Adherence (%)	80.09±4.87	88.75±10.03	98.5	<.001	0.232
Treatment adherence ≥85%	0 (0%)	41 (80.4%)	—^g^	—	—

a PANAS: Positive and Negative Affect Schedule.

b DASS-21: Depression Anxiety and Stress Scale-21.

c MHC-SF: Mental Health Continuum Short Form.

d GSES: Generalized Self-Efficacy Scale.

e HrQoL: health-related quality of life.

f QoLISSY: Quality of Life in Short Stature Youth.

g Not applicable.

#### Mental Well-Being of Parents

**Figure 2.**
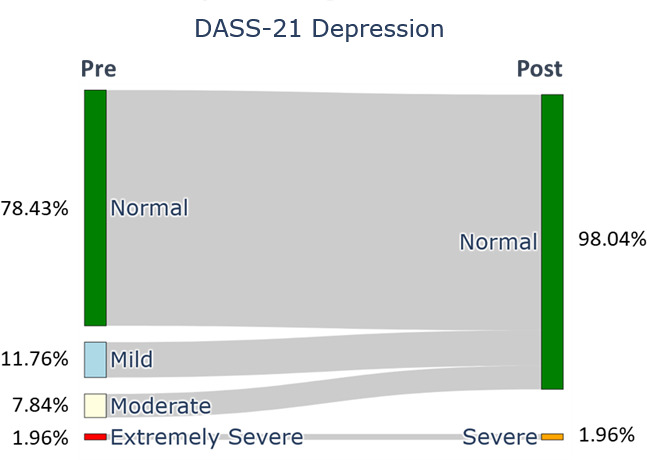
Depression symptoms of family caregivers (baseline and 12 wk).

**Figure 3.**
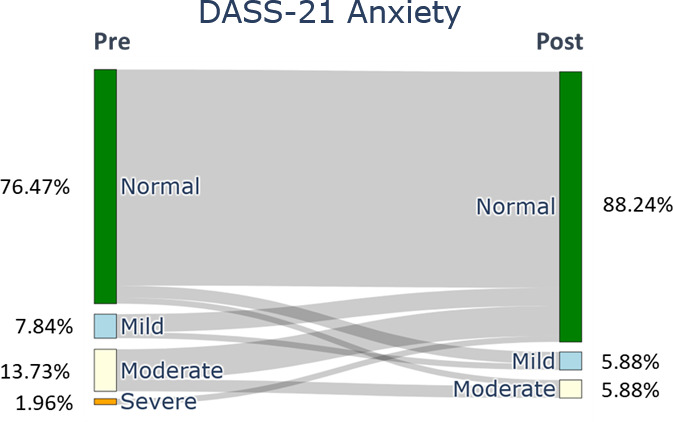
Stress symptoms of family caregivers (baseline and 12 wk).

**Figure 4.**
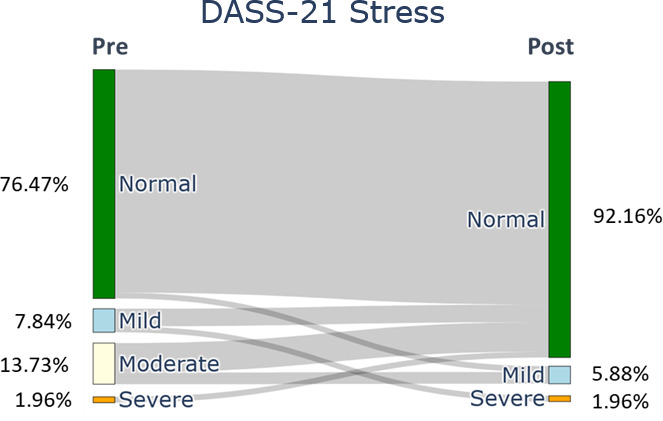
Stress symptoms of family caregivers (baseline and 12 wk).

After the ACDP, the participants showed a significant difference in all the QoLISSY subscales except for the emotional (*P*=.28) scales. Meaningful results were found in the physical and social dimensions and in the child’s future perception, coping, treatment, height-related beliefs, and effects of the child’s short stature on the family scale. Concerning the mental health symptoms, the depression (Figure 2), anxiety (Figure 3), and stress (Figure 4) symptoms measured by the DASS-21 were reduced after the 3-month digital intervention.

Finally, the use of the ACDP was associated with a significant increase in the adherence rate (*P*<.001). At the baseline, all the families had suboptimal adherence (below 85%); after the intervention, 75% (n=38) of the families reached optimal levels of adherence. The QoLISSY item regarding perceived pain of injection was also reduced, which might explain that improved social well-being and reduced anxiety and stress were associated with reduced perceived pain from injection and overall contributed to improved adherence.

#### Adult Growth Prediction

In this study, growth data (height) have been analyzed post hoc for studying the impact of the intervention on patients treated with GH in a post hoc explorative analysis phase. In this sense, patients’ growth estimations are calculated by means of deviation from baseline children’s growth charts, based on the participants’ adherence levels to GHt. Specifically, measurements of patients’ height were collected in four fixed time points: (1) 6 months before treatment, (2) start of treatment, (3) start of the study, and (4) 6 months after the start of the study. Finally, another important variable ΔHSDS (change in height standard deviation score) that has been computed is the change in height deviation values between consecutive time points. The impact of the intervention in the growth status was analyzed by focusing on the patients who switched from medium to high adherence (~70% of the participants). Thus, assuming that without participation in the study, the patients’ adherence levels would have been medium, and the overall effect on mean ΔHSDS was computed by comparing the 48-month estimations of medium-only (hypothetical) cases with the real situation (70% high and 30% medium adherence, based on data analysis).

The average estimated ΔΗSDS value for patients with medium adherence was 0.9, while that of the real (70% high and 30% medium adherence) cases was 1.04. Hence, an increase of 0.14 was observed in terms of ΔHSDS, meaning that the average participant is going to grow more thanks to the intervention. In other words, the increase in ΔHSDS indicates a reduction in the height gap between the patient’s actual growth and the expected growth based on baseline charts. This suggests improved growth outcomes due to better adherence to the treatment.

## Discussion

### Principal Results

The management of children undergoing GHt has been reported to be burdensome [14]; specifically, it can be a time-consuming and exhausting process as family caregivers need to (1) ensure that their children receive the injections at the right time and at the correct dosage while providing emotional support and (2) address any mental health issues that may arise. Family caregivers’ fatigue and stress can negatively influence treatment adherence and the child’s health [14,15]. This research contributes to the emerging work in supporting family caregivers of children undergoing GHt using digital tools [28] by showing that the ACDP can effectively empower family caregivers of children with GHD, including improvements in the well-being of both parents and children while promoting self-management of the condition.

Participants have reported a better quality of life and general health, as well as improvements in key points such as cognitive symptoms, mood, and emotional and social well-being. Most families involved in the study achieved optimal levels of adherence after the study. We found a statistically significant increase in positive affect (from 30.78, SD 7.27 at baseline to 36.53, SD 8.65 at the 3-month follow-up) with a statistically significant decrease in negative affect (from 18.98, SD 6.02 to 14.57, SD 4.18), which was measured using the PANAS. Also, the MHC-SF revealed a statistically significant increase in general well-being (from 0.62, SD 0.17 to 0.69, SD 0.21), social well-being (from 0.52, SD 0.19 to 0.65, SD 0.22) and general self-efficacy (from 31.31, SD 5.51 to 33.43, SD 5.31), with an increase in emotional (from 0.67, SD 0.20 to 0.71, SD 0.20), and psychological well-being (from 0.67, SD 0.18 to 0.72, SD 0.23). KIDSCREEN-10 revealed a statistically significant increase in HrQoL (from 49.19, SD 10.82 at baseline to 60.89, SD 16.86 at the 3-month follow-up) and a statistically significant increase in general health (3.43, SD 0.81 to 4.22, SD 0.81). Regarding the quality of life of youths with short stature, we found a statistically significant increase in scores on the physical scale (from 75.57, SD 19.51 to 81.94, SD 18.38), social scale (from 69.83, SD 20.35 to 77.72, SD 18.15), coping scale (from 40.98, SD 17.25 to 48.48, SD 21.32), height-related beliefs scale (from 8.31, SD 25.96 to 66.30, SD 28.20), treatment scale (from 53.54, SD 17.77 to 67.37, SD 21.93), and child’s future scale (from 73.63, SD 25.57 to 82.65, SD 17.98), as well as an increase in HrQoL (from 74.94, SD 15.93 to 80.67, SD 14.46), and emotional scale (from 79.41, SD 15.07 to 82.35, SD 13.53). Lastly, we found statistically significant increases in growth rate on the child’s short stature on the family’s section of the QoLISSY (from 68.45, SD 18.94 to 77.72, SD 18.20) and treatment adherence (from 80.27, SD 4.77 to 88.98, SD 9.84).

Our findings show that at baseline, 21.56% (n=11) of caregivers reported depression symptoms, categorized as mild (11.76%, n=6), moderate (7.84%, n=4), and extremely severe (1.96%, n=1). Post intervention, depression was reduced to only severe (1.96%, n=1). Anxiety levels at baseline were mild (7.84%, n=4), moderate (13.73%, n=7), and severe (1.96%, n=1) for 23.53% (n=12) of caregivers. After the intervention, 11.76% (n=6) of caregivers reported mild (5.88%, n=3) or moderate (5.88%, n=3) anxiety levels. Stress symptoms also improved; initially, 23.5% (n=12) reported mild (7.84%, n=4), moderate (13.72%, n=7), and severe (1.96%, n=1) stress. Following the intervention, stress symptoms reduced to 7.84% (n=4), with only mild (5.88%, n=3) and severe (1.96%, n=1) symptoms remaining.

### Limitations

This is a local study conducted in Zaragoza (Spain) with a small sample and no comparison arm. Because of inclusion/exclusion criteria, people with low digital literacy were not able to participate. Although the general prevalence of GHD is higher in boys, most caregivers participating in this study had daughters who have GHD; thus, parents of boys with GHD might have been underrepresented.

### Comparison With Prior Work

The findings from this study align with and extend previous research on digital interventions aimed at supporting family caregivers of children undergoing GHt. Prior studies have highlighted the significant burden on family caregivers managing GHt, including the physical, emotional, and psychological stress that can negatively impact treatment adherence and the overall health of the child [13]. The ACDP demonstrates notable advancements in this area by providing a comprehensive digital health intervention that effectively supports both the physical and mental well-being of caregivers [33].

Previous research by Arriba et al [34] suggested that mobile solutions could potentially improve GHt adherence by addressing the emotional states of parents and caregivers. Our study, albeit observational, provides further evidence that digital tools appear to significantly enhance adherence rates by improving caregivers’ psychological states. In particular, our study revealed a statistically significant increase in treatment adherence from baseline (below 85%) to postintervention levels, with 75% of families reaching optimal adherence.

Furthermore, the mental well-being of caregivers showed substantial improvement, a finding consistent with earlier work by de Arriba et al [34], which highlighted the positive impact of the ACDP on caregivers’ mental health. Our findings show that at baseline, 21.56% (n=11) of caregivers reported depression symptoms, categorized as mild (11.76%, n=6), moderate (7.84%, n=4), and extremely severe (1.96%, n=1). Anxiety and stress levels also showed marked reductions, aligning with the findings from the study by Cervera-Torres et al [22], which emphasized the importance of digital health support for emotional and self-management needs.

Moreover, our study’s comprehensive assessment of quality of life and well-being metrics—using tools such as the PANAS, MHC-SF, and KIDSCREEN-10—demonstrated significant improvements in cognitive symptoms, mood, emotional and social well-being, and general health. These results further extend the insights from the study by Savage et al [13], which discussed the transformative potential of patient-generated data in integrated care models for GHt. The significant increases in positive affect, general well-being, social well-being, and self-efficacy among participants underscore the holistic benefits of digital health interventions.

### Conclusions

In conclusion, the ACDP showed favorable acceptance for family caregivers of children undergoing GHt. It is a promising tool and has been demonstrated to add value to the patient and caregiver journey by improving GHD management while supporting the overall well-being of family caregivers. It has helped parents improve their mental well-being as well as treatment adherence. This study provides insights into how digital interventions can better support families of children undergoing GHt.

## Abbreviations

ACDP: Adhera Caring Digital Program
DASS-21: Depression Anxiety and Stress Scale-21
DH: digital health
GH: growth hormone
GHD: growth hormone deficiency
GHt: growth hormone therapy
GSES: Generalized Self-Efficacy Scale
HrQoL: health-related quality of life
ISO: International Organization for Standardization
MHC-SF: Mental Health Continuum Short Form
PANAS: Positive and Negative Affect Schedule
QoLISSY: Quality of Life in short stature youths
ΔHSDS: change in height standard deviation score
